# Impaired pulmonary and muscle function during moderate exercise in female patients recovered from SARS-CoV-2

**DOI:** 10.1038/s41598-022-24941-9

**Published:** 2022-12-04

**Authors:** Eulogio Pleguezuelos, Amin Del Carmen, Eva Moreno, Pilar Ortega, Alejandro Robles, Mateo Serra-Prat, Marc Miravitlles, Joan Carles Yebenes, Manuel V. Garnacho-Castaño

**Affiliations:** 1 Physical Medicine and Rehabilitation Department, Hospital de Mataró, Barcelona, Spain; 2 Department of Experimental Science and Healthcare, Faculty of Health Sciences, Universitat Pompeu Fabra, Barcelona, Spain; 3 Physical Medicine and Rehabilitation Department, Hospitalet General Hospital, L’Hospitalet de Llobregat, Barcelona, Spain; 4 Pneumology Department, Hospital de Mataró, Barcelona, Spain; 5 Research Unit, Consorci Sanitari del Maresme, Mataró, Barcelona, Spain; 6 Pneumology Department, Hospital Universitari Vall d’Hebron, Vall d’Hebron Institut de Recerca (VHIR), Barcelona Hospital Campus, CIBER de Enfermedades Respiratorias (CIBERES), Barcelona, Spain; 7 Critical Care Department, Hospital de Mataró, Barcelona, Spain; 8 Campus Docent Sant Joan de Déu, Fundación Privada, Universitat de Barcelona, Carrer de Miret i Sans, 10, 08034 Barcelona, Spain

**Keywords:** Physiology, Respiration, Respiratory tract diseases

## Abstract

This study aimed to assess pulmonary and muscle dysfunction by analyzing the slow component of oxygen uptake (VO_2SC_), and mechanical and ventilatory efficiency in adult women recovered from the severe acute respiratory syndrome coronavirus type II (SARS-CoV-2) during a constant load test. 32 women (N = 17 patients with SARS-CoV-2; N = 15 control group) performed two cardiopulmonary exercise tests (CPX) on a cycle ergometer. In the first test, the participants performed incremental CPX until extenuation. In the second test the participants performed a 10-min CPX at a constant load intensity (watts) corresponding to the first ventilatory threshold. There was a 48–72 h rest period between the two tests. There was a significant increase in the VO_2SC_ in the patients recovered from SARS-CoV-2 (160.4 ± 60 mL min^−1^) in comparison with the healthy participants (59.6 ± 65 mL min^−1^) (*P* < 0.001). Mechanical efficiency significantly decreased in patients recovered from SARS-CoV-2 compared to the control group (*P* = 0.04). Ventilatory inefficiency significantly increased in the patients recovered from SARS-CoV-2 compared with the control group (*P* < 0.001). Adult women recovered from SARS-CoV-2 infection have important pulmonary and muscular dysfunction and fatigue which contributes to increasing the VO_2SC_ and reducing mechanical and ventilatory efficiency during mild-moderate exercise at a constant load.

## Introduction

Multisystemic cardiopulmonary and muscular involvement produces fatigue, pain and muscle weakness as the symptomatology associated with severe acute respiratory syndrome coronavirus type 2 (SARS-CoV-2)^1,2^. There is an evident female preponderance in the development of fatigue although all patients diagnosed with SARS-CoV-2 will require fatigue screening^3^.

The assessment of pulmonary oxygen uptake kinetics has been suggested to determine fatigue and a reduced exercise tolerance in cardiovascular and respiratory diseases^4,5^. Specifically, the pulmonary VO_2_ tends to rapidly increase from the onset of exercise until approximately 3 min during prolonged cardiopulmonary exercise test (CPX) at a constant-load. After 3 min, the VO_2_ slowly increases for a given production of power, implicating sustained lactic acidosis and surpassing the fundamental component of the beginning of exercise^6^ until a delayed stable state is acquired or maximum VO_2_ is reached^7^. This VO_2_ response is known as the slow component of VO_2_ (VO_2SC_)^6^. The evaluation of VO_2SC_ has been used as an indicator of the appearance of fatigue limiting exercise tolerance in respiratory diseases^5,8^.

A key parameter associated with the behavior of the VO_2SC_ during exercise is mechanical efficiency (ME), which estimates the effects of alkalinization of the blood in gradual loss of muscle efficiency^6^. Patients with reduced ME are less muscularly efficient and can manifest limited capacity for physical activity^9^. The association between the VO_2SC_ and loss of muscle strength during exercise propose a common physiological mechanism between loss of muscle efficiency and skeletal muscle fatigue^10^.

Evaluation of ventilatory efficiency is another relevant parameter in the diagnosis of CPX in cardiorespiratory diseases^11,12^, essentially to understand the relationship of ventilation (VE) and perfusion in the lungs. Evaluation of the VE and carbon dioxide production slope (VE∙VCO_2_^–1^ slope) is considered as the reference method for predicting mortality, ventilatory inefficiency and exercise tolerance in cardiorespiratory diseases^11–13^.

The muscle weakness and fatigue experienced in patients recovered from SARS-CoV-2 suggests that marked pulmonary and muscle dysfunction alters VO_2SC_ kinetics, reducing ME and increasing ventilatory inefficiency during exercise. However, to our knowledge, this hypothesis has not been scientifically corroborated.

This study aimed to evaluate pulmonary dysfunction and muscle fatigue by analyzing the VO_2SC_ kinetics, ME and ventilatory efficiency during a constant-load test on a cycle ergometer in women who have had coronavirus disease 2019 (COVID -19). We hypothesize that the VO_2SC_ increases and mechanical and ventilatory efficiency decrease to a greater extent in adult women recovered from COVID-19 compared to healthy adult women during a constant-load test on a cycle ergometer.

## Methods

### Participants

Seventeen adult women with COVID-19 and fifteen healthy adult women (control group) were recruited for the study. Information on previous medical history was collected. The adjusted morbidity group, which classifies morbidity according to data codified by the health care system, was obtained for all the participants in both groups^14^.

In the patients with COVID-19, we collected data related to hospital admission and determined the Acute Physiology and Chronic Health Disease Classification System II (APACHE II) as a predictive system of disease severity and outcome of patients in intensive care units^15^. The female patients recovered from COVID-19 included in this study were considered healthy prior to SARS-CoV-2 infection. The main symptoms of female patients recovered from COVID-19 were fatigue and dyspnea. These patients were previously evaluated by the pulmonology, cardiology, and internal medicine services to rule out any type of cardiac or pulmonary pathology that could be the cause of their symptoms (fatigue and dyspnea). To avoid selection bias, patients with any pathology (except COVID-19) that could condition performance in CPX were excluded from the study. The group of healthy adult women did not suffer from any type of disease.

The exclusion criteria were: severe neurological disease, active oncologic disease, neuromuscular and/or orthopedic disorders impeding normal performance of CPX; the absence of signed informed consent.

Inclusion criteria were: age > 18 years; molecular (RT-PCR) diagnosis of SARS-CoV-2 infection; hospitalized and non-hospitalized adult female recovered from COVID-19; healthy adult female who did not have COVID-19 at any time (control group).

The sample size estimation was performed with α = 0.05 (5% type I error probability) and 1 − β = 0.80 (80% power). For this study, a total of 15 adult female survivors of COVID-19 were required to detect differences between both experimental conditions.

The study was approved by the Committee of Ethics and Investigation of the hospital (Code: 89/20). The study protocol was performed according to the principles of the Declaration of Helsinki. Written informed consent was collected from all participating women.

### Incremental and constant cardiopulmonary test

Prior to the CPX tests, the participants underwent a spirometry test. Patients with COVID-19 performed the incremental and constant CPX on a cycle ergometer with an electromechanical brake (Ergoline900S, Ergoline GmbH, Bitz, Germany). For security reasons, the control group performed the CPX on a cycle ergometer with an electromechanical brake (Ergoselect 100P, Ergoline GmbH, Bitz, Germany). There was a 48–72 h rest period between the two tests. The assessment of patients with COVID-19 was carried out 6–8 weeks after discharge from hospital.

The protocol of incremental CPX until extenuation was performed individualized and adapted to all the participants with gradual increments of 5, 10, 15 or 20 W min^−1^. In the incremental CPX, the first ventilatory threshold (VT_1_) was determined using the method of ventilatory equivalents described by Skinner et al.^16^ in which the VT_1_ corresponds to an increase of VE∙VO_2_^–1^ without an increase in VE∙VCO_2_^–1^, coinciding with an increase of end*-*tidal expired partial oxygen pressure *(*PetO_2_).

The protocol of the constant CPX was individualized with 10 min on a cycle ergometer at an intensity corresponding to the VT_1_. The patients were told to maintain a constant similar pedaling cadence (50–80 rpm) in each test. Throughout the whole test the patients underwent continuous 12-lead electrocardiographic monitoring.

An open circuit breath-by-breath Ultima CardiO2 gas analyzer was used for COVID-19 patients (Medical Graphics Corporation, St. Paul, Minnesota, USA) an Ergostik analyzer was used for the control group (Geratherm Respiratory, Bad Kissingen, Germany).

### Slow component of VO_2_ assessment

During the constant-load test at an intensity of the VT_1_, pulmonary VO_2_ kinetics were evaluated. Data of pulmonary VO_2_ were registered during 2 min prior to initiation of the constant test (basal status). The basal VO_2_ (VO_2B_) was considered as the mean during the last 60 s prior to beginning the test. The fundamental kinetics (Phase II) of VO_2_ were determined using previously described criteria and were adjusted to a monoexponential function^17^: VO_2_(*t*) = VO_2B_ + ∆VO_2FP_ × (1 – *e*^– *(t*–TR/τ^), where VO_2_(*t*) is the value of pulmonary VO_2_ at any time *t* of the VO_2_ kinetics; VO_2B_ is the of basal VO_2_ value; ∆VO_2FP_ is the increase in VO_2_ above the baseline reference values and determines the width of the fundamental phase, and τ is the constant of time of the fundamental phase. TR is the time delay. The exponential region of each participant was individually adjusted^18^. To determine the VO_2SC_ (Phase III), the data of pulmonary VO_2_ was registered with an average interval of 3 s. Finally, the VO_2SC_ was determined in each participant: ∆VO_2SC_ = VO_2peak_ – (VO_2B_ + ∆VO_2FP_)^18^.

### Evaluation of mechanical efficiency

The ME during the constant-load test was estimated as follows. The corresponding energy expenditure (EE) was calculated. The net VO_2_ was obtained by subtracting resting VO_2_ from the total VO_2_ throughout the exercise. The net EE was calculated as: (4.94 × RER + 16.04) × (VO_2_net, in ml min^−1^) × 60^–1^^19^. The ME was also calculated in net terms: work converted in watts × net EE × 100^–1^^20^.

### Evaluation of ventilatory efficiency

In the incremental and constant CPX ventilatory efficiency was determined as the slope of the relationship between VE and VCO_2_ (VE∙VCO_2_^–1^ slope)^4,21^. In the incremental CPX, the VE∙VCO_2_^–1^ slope was calculated from the beginning of the incremental exercise until the time of ventilatory compensation by linear regression^22,23^.

### Statistical analysis

The Shapiro–Wilk test was used to verify normal distribution of the data expressed as mean and the standard deviation (SD) and the mean and the corresponding 95% confidence intervals (95%CI). Analysis of variance was performed to determine differences between the two study groups in the normal variables and the Mann–Whitney U test was used for non-normal variables.

To determine the magnitude of the difference in the variables analyzed, the *d* of Cohen was used to test the effect size, which was defined as large with *d * ≥ 0.80, moderate with *d * ≥ 0.40 and small with *d* < 0.40^24^. A Pearson correlation coefficient was applied to determine significant relations among the variables analyzed. Statistical significance was set as p < 0.05. The SPSS software package version 25.0 for Mac (SPSS Inc., Chicago, IL, USA) was used to perform the statistical analyses.

### Ethics statements

The study was approved by the Committee of Ethics and Investigation of the Mataró hospital (Code: 89/20). The study protocol was performed according to the principles of the Declaration of Helsinki, Good Practice Guidelines and local applicable regulations.

### Consent to participate

All participants signed for written informed consent.

## Results

Table 1 shows the main characteristics of the two study groups at baseline, the spirometry and incremental CPX results. The most relevant characteristics of the patients with SARS-CoV-2 are shown in Table 2.

**Table 1:** Descriptive characteristics of the patients with SARS-CoV-2 and control group.

	CG	SARS-CoV-2	p-value
Age (years)	45.7 (7.1)	46.4 (12.6)	0.841
Weight (kg)	65.3 (13.5)	74.5 (15.5)	0.061
Height (m)	1.6 (0.1)	1.6 (0.1)	0.224
BMI (kg min^−2^)	24.9 (5.8)	29.4 (5.9)	**0.008**
**Adjusted morbidity groups (%)**			
Basal risk	100%	29.4%	
Low risk	0%	41.2%	
Moderate risk	0%	17.6%	
High risk	0%	11.8%	
Very high risk	0%	0%	
FVC (L)	3.6 (0.3)	3.2 (0.7)	0.088
FVC (%)	100.5 (11.1)	86.4 (13.5)	**0.005**
FEV1 (L)	2.9 (0.3)	2.7 (0.6)	0.079
FEV1 (%)	104.9 (12.2)	91.4 (15.7)	**0.016**
FEV1/FVC (%)	83 (5.7)	83.2 (7.5)	0.932
VO_2peak_ (mL kg m^−1^)	24.2 (5.3)	16.7 (5.4)	**0.001**
Peak power (W)	127.9 (32.6)	95.2 (31.5)	**0.008**
Power at VT_1_ (W)	57.7 (18.2)	35.4 (18.6)	**0.002**

Data are provided as mean and standard deviation (SD).

*BMI* body mass index, *CG* control group, *FEV1* forced expiratory volume in the first second, *FVC* forced vital capacity, *SARS-CoV-2* severe acute respiratory syndrome coronavirus type 2, *VO*_*2*_*peak* peak oxygen uptake, *VT*_*1*_ first ventilatory threshold.

Significant values are given in bold.

**Table 2:** Clinical characteristics of the patients with SARS-CoV-2.

Hospital admission, yes (%)	8 (47.1%)
ICU admission, yes (%)	5 (29.4%)
ICU admission days	14.3 (7.3)
Days of hospital admission	25.4 (13.8)
Days of mechanical ventilation	8.5 (0.7)
Tracheostomy (no, %)	8 (100%)
Prone positioning (yes, %)	14 (53.8%)
Apache II/ICU admission	10.5 (4)

*ICU* intensive care unit.

### Slow component of VO_2_

**Figure 1:**
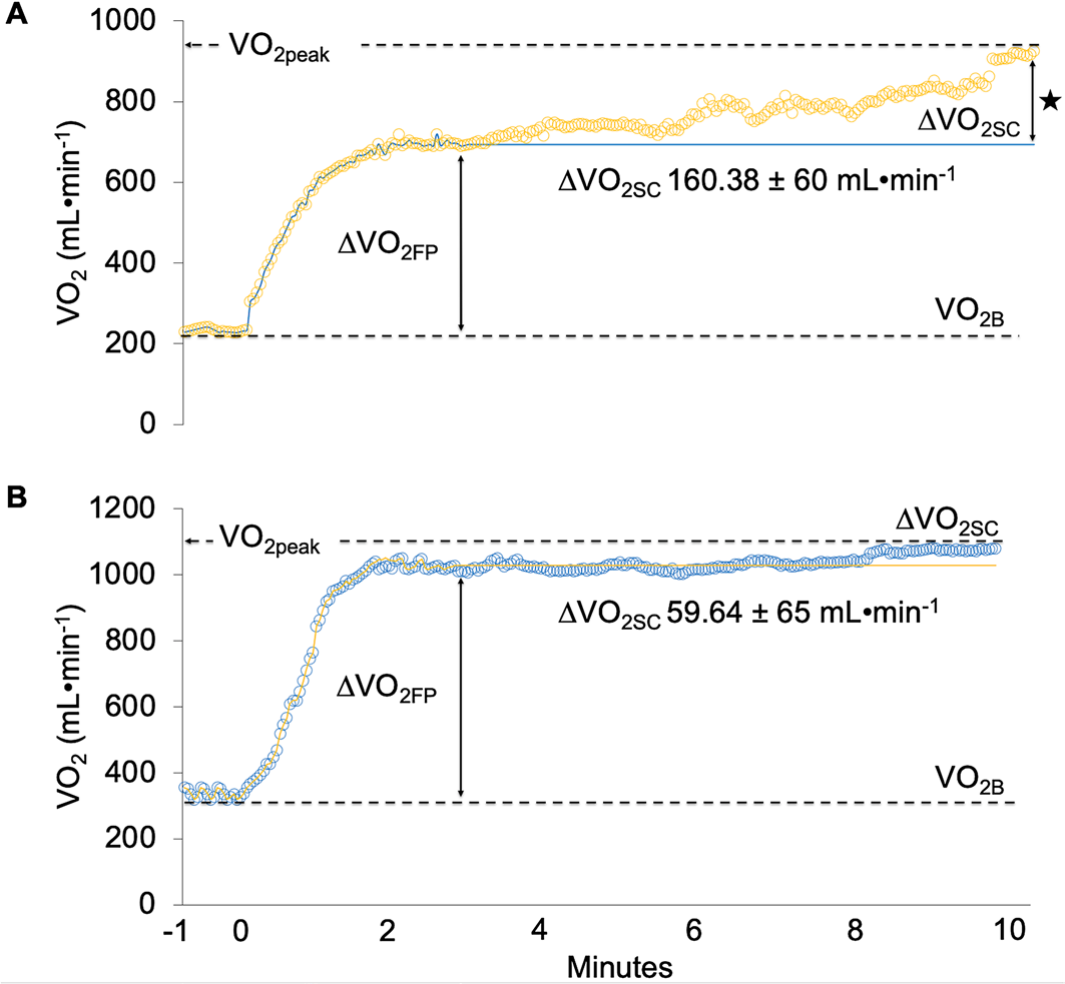
Kinetics of oxygen consumption (VO_2_) during the constant load test at the intensity of the first ventilatory threshold (VT_1_). (**A**) In patients with SARS-CoV-2. (**B**) Control group. The orange line (circles) corresponds to the data collected by the patients with SARS-CoV-2. The blue line (circles) corresponds to the data collected by the control group. The solid blue line corresponds to the assumed steady state of expected VO_2_ at an intensity of VT1 in the patients with SARS-CoV-2. The solid orange line corresponds to the assumed steady state of expected VO_2_ at an intensity of VT1 in control group. Data for VO_2_ kinetics are provided as the mean of all participants. ★ Patients with SARS-CoV-2 significantly increased the slow component of VO_2_ (VO_2SC_) compared to the control group (p < 0.001). *∆VO*_*2FP*_ increase in oxygen uptake in the fundamental phase, *VO*_*2B*_ baseline oxygen consumption.

**Figure 2:**
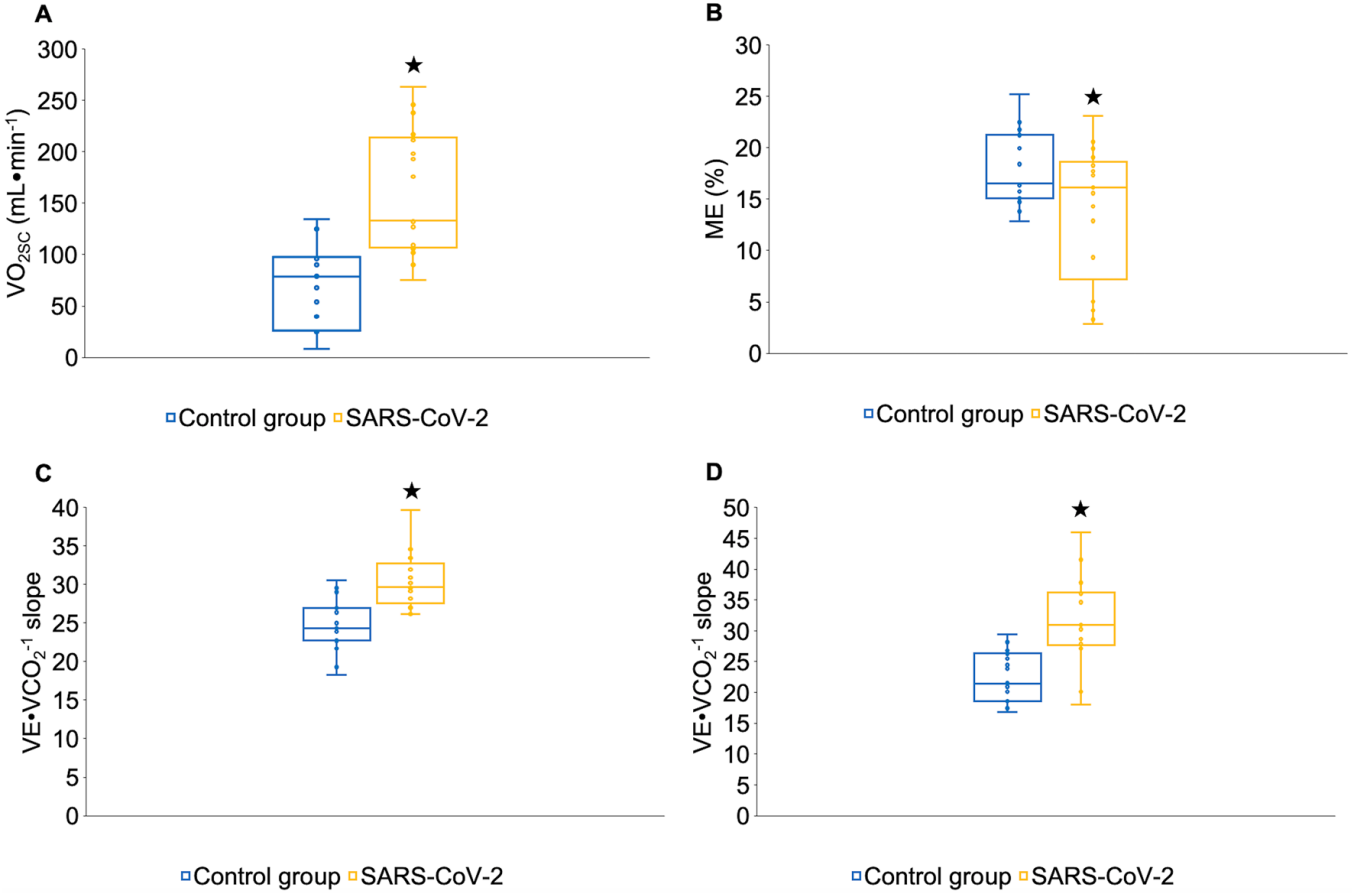
Differences between the patients with SARS-CoV-2 and the control group. (**A**) Slow component of VO_2_ (VO_2SC_) during the constant-load test at the intensity of the first ventilatory threshold (VT1). (**B**) Mechanical efficiency (ME) during the constant-load test at the intensity of the VT1. (**C**) Ventilatory efficiency during incremental cardiopulmonary test. (**D**) Ventilatory efficiency during the constant-load test at the intensity of the VT1. Data are given as mean and error bars correspond to 95% confidence intervals (95% CI). ★ The patients with SARS-CoV-2 increased the VO_2SC_ (p < 0.01) and the VE∙VCO_2_^–1^ slope (p < 0.001), and decreased the mechanical efficiency compared to the healthy control group (p < 0.05).

There was a significant increase in the VO_2SC_ in the patients with SARS-CoV-2 in comparison with the healthy participants (F = 20.24, *P* < 0.001, *d* = 1.53) (Figs. 1 and 2A).

### Mechanical and ventilatory efficiency

The ME significantly decreased in patients with SARS-CoV-2 with respect to the control group (F = 4.24, *P* = 0.04, *d* = − 1.10) (Fig. 2B).

In relation to ventilatory efficiency, the VE∙VCO_2_^–1^ slope significantly increased in the patients with SARS-CoV-2 in comparison with the control group during the incremental test (F = 16.60, *P* < 0.001, *d* = 1.67) (Fig. 2C) and in the constant load test (F = 19.23, *P* < 0.001, *d* = 2.21) (Fig. 2D).

**Figure 3:**
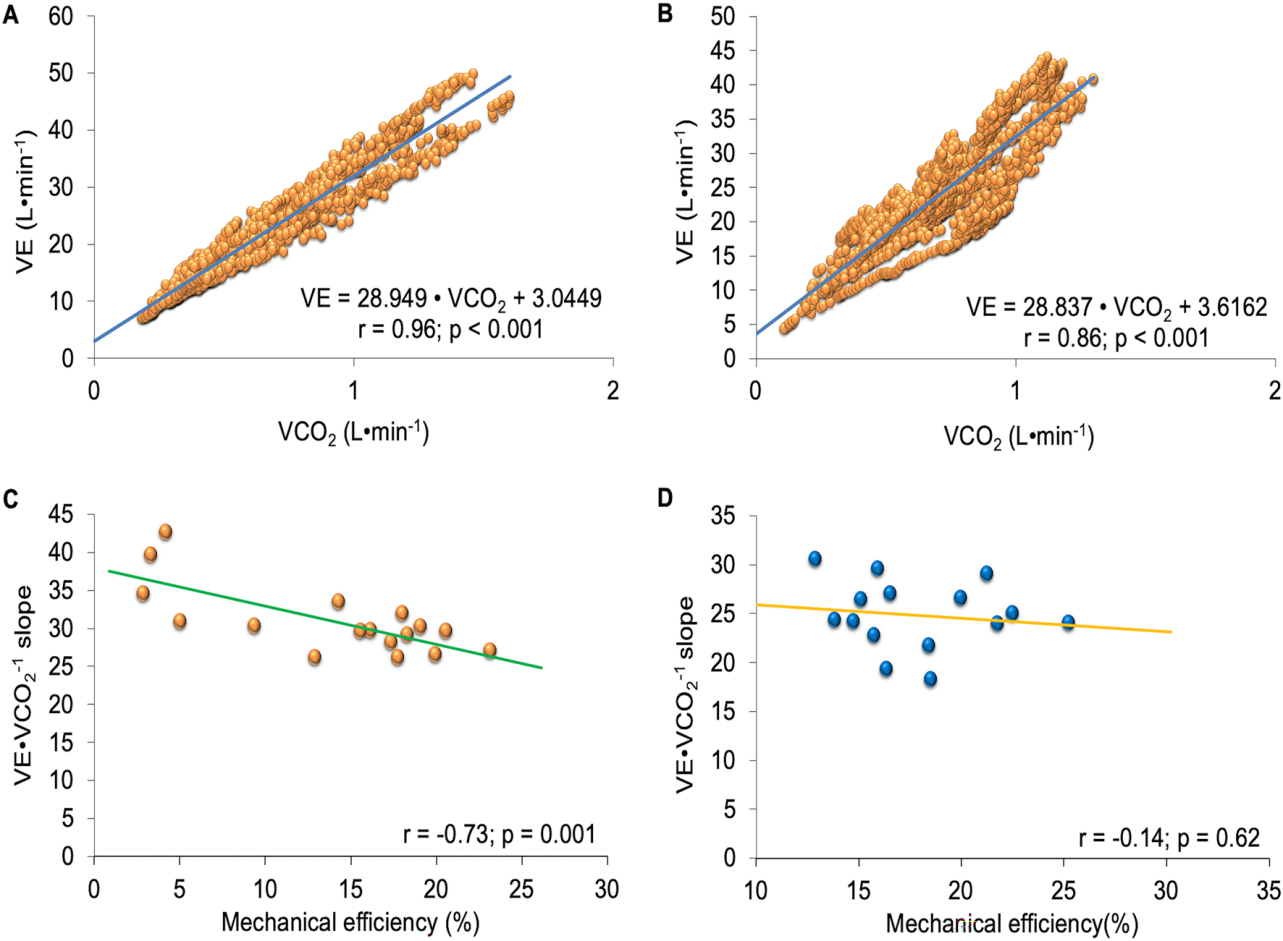
Correlations between variables: (**A**) VE∙VCO_2_^–1^ slope during incremental cardiopulmonary test in patients with SARS-CoV-2. (**B**) VE∙VCO_2_^–1^ slope during the constant load test at the intensity of the first ventilatory threshold (VT_1_) in patients with SARS-CoV-2. (**C**) Mechanical efficiency during the test at constant load at VT_1_ and the VE∙VCO_2_^–1^ slope during the incremental test in patients with SARS-CoV-2. (**D**) Mechanical efficiency during the test at constant load at VT_1_ and the VE∙VCO_2_^–1^ slope during the incremental test in the control group.

Highly significant correlations were observed between VE and VCO_2_ during the incremental test (r = 0.96; *P* < 0.001) (Fig. 3A) and the constant load test (r = 0.86; *P* < 0.001) (Fig. 3B) in the SARS-CoV-2 patients. In addition, there was a significant inverse correlation between the ventilatory efficiency obtained in the incremental test and the ME achieved during the constant load in the patients with SARS-CoV-2 (r = − 0.73; *P* = 0.001) (Fig. 3C). This correlation was not confirmed in the control group (*P* > 0.05) (Fig. 3D).

## Discussion

To the best of our knowledge, this is the first study to assess the VO_2SC_, ME and ventilatory efficiency in adult women recovered from SARS-CoV-2 during a constant-load test at VT_1_ intensity. The most relevant finding of this study was that patients recovered from SARS-CoV-2 presented an elevated VO_2SC_, deteriorated ME and significant ventilatory inefficiency in comparison with the control group of healthy women. Furthermore, the patients with COVID-19 with better ventilatory efficiency showed increased ME during the constant-load test. We can state that adult women recovered from SARS-CoV-2 suffered a greater impaired pulmonary and muscle function during moderate exercise than healthy adult women.

No previous study has assessed the VO_2SC_, ME and ventilatory efficiency during a constant-load test in adult women recovered from COVID-19. Therefore, the discussion of the results was based on data reported by other studies in diseases with impaired pulmonary and muscle function during exercise.

We observed a substantial increase in the VO_2SC_ in patients with COVID-19 compared to the control group. This elevation was especially notable if it is taken into account that the exercise performed at a constant-load at an intensity of the VT_1_ should stimulate a stable state or plateau in VO_2_ behavior^4^. While a stable state was observed in the VO_2_ kinetics in healthy women, in the patients with COVID-19 there was a progressive increase in the VO_2SC,_ especially at the end of the test.

In comparison with our study, Chiappa et al. reported a higher VO_2SC_ in both patients with chronic obstructive pulmonary disease (COPD) (202 ± 99 mL min^−1^) and in the control group (185 ± 89 mL min^−1^)^5^. Similar results were described by Laveneziana et al. in patients with COPD (191 ± 131 mL min^−1^)^25^. The differences observed with respect to our study could be attributed to the constant-load tests being more intense in both studies in terms of relative intensity (~ 75% of the peak load). The patients with COVID-19 and the healthy participants pedaled at a load intensity of ~ 37% and ~ 45%, respectively, of the peak power. The intensity of relative load (%) performed by the patients with SARS-CoV-2 was approximately half that reported in the above mentioned studies^5,25^.

The physiological mechanisms producing the increase in VO_2SC_ are uncertain, especially in cardiorespiratory diseases since the power or the equivalent load at the intensity of the lactate threshold or VT_1_ means, in theory, the greater power or load the increase in the VO_2SC_ does not produce. Elevated VO_2SC_ response only occurs at intensities above the lactate threshold and the VT_1_^4,26^. It seems that a mild-moderate intensity such as VT_1_ conditions the behavior of the VO_2SC_ in patients recovered from COVID-19 to a greater extent than in other diseases with cardiorespiratory involvement at a similar or event lower relative intensity^8,27^. Studies evaluating the kinetics of VO_2_ at lower and higher intensities are needed to corroborate these suppositions in patients recovered from COVID-19.

As mentioned previously, the trend of the VO_2_ kinetics observed in patients recovered from COVID-19 demonstrated a significant increase, especially at the end of the test. It can be assumed that this increase in VO_2SC_ was associated with a progressive decline in muscular efficiency and^28^, consequently, with the appearance of metabolic fatigue in response to the transition towards a more anaerobic metabolism^28–30^. This muscular inefficiency induced by the VO_2SC_ could be related to forced delayed recruitment of less efficient motor units (type II) from the oxidative point of view, simply to compensate the production of attenuated strength in motor units already active during pedaling. In this physiological and metabolic context, preferential glycogen depletion of type I muscle fibers would be produced^31^. The recruitment of type II motor units has been suggested as a more acceptable explanation to understand the increase of the VO_2SC_^6,7^. It is likely that 10 min of exercise at a VT_1_ intensity stimulated premature and progressive recruitment of less efficient motor units in the patients recovered from SARS-CoV-2.

The ME could, at least in part, explain the behavior of the VO_2SC_ estimating the losses of muscular efficiency^6^. The results of ME observed in the constant-load test in female patients recovered from COVID-19 (~ 14% of ME at 37% of peak power) were lower than those achieved by the control group (~ 18% at 45% of peak power), and those reported by Baarends et al. who described net mean values of 15.5% in patients with COPD at an intensity corresponding to 50% of the peak load during a constant-load test^32^. Similar values of 16% were reported in patients with mild and moderate COPD at an intensity of 50% of the maximum power during a constant-load test^33^. In any case, this demonstrates that values less than 17% in patients with COPD are indicative of reduced ME^32^.

We previously demonstrated that patients with COVID-19 showed a decreased delta efficiency similar to that observed in patients with COPD and ischemic heart disease during an incremental test^34^. Other studies with patients recovered from the acute phase of COVID-19 have shown that patients with decreased exercise capacity showed a higher degree of deconditioning and lower levels of performance and earlier termination, with a lower work during a CPX^35^. Probably, lower ME and deconditioning observed in patients recovered from COVID-19 could be related with an impaired O_2_ extraction and use caused by a direct effect of viral load on muscle tissue^36^. It seems that the reduction in ME could be related to the energy expenditure in the skeletal muscle. Layec et al. proposed a greater ATP cost during muscle contraction as the main cause of more reduced ME in patients with COPD^37^. This increase in energy expenditure is generally associated with an increase in the proportion of type II muscular fibers during exercise in patients with COPD^38^. The higher forced recruitment of type II fibers could justify, at least in part, an impaired extraction and use of O_2_, decreasing ME in female patients recovered of COVID-19 during a constant-load exercise of light-moderate intensity.

Other studies have proposed unusually low levels of fatty acid β-oxidation and altered lactate production by skeletal muscle as a functional limitation in patients with post-acute sequelae of SARS-CoV-2 infection^39^. In theory, a high blood lactate concentration at lower exercise intensities suggests mitochondrial dysfunction^40^. Mitochondrial dysfunction has been evidenced as an inherent cause in understanding the pathogenesis of post-acute sequelae of SARS-CoV-2 infection in patients with preserved pulmonary and cardiac function^39^.

Patients with a severely deteriorated ME are characterized by increased ventilatory response to exercise and by a reduction of the maximum capacity to perform exercise^32^. Consequently, the efficiency of pulmonary gas exchange is reduced, producing unadjustment between VE/perfusion, thereby increasing ventilatory inefficiency^4^. In patients recovered from SARS-CoV-2, the VE∙VCO_2_^–1^ slope increased demonstrating reduced ventilatory efficiency in comparison with the control group. This pulmonary dysfunction could affect perfusion in active muscle reducing tolerance to exercise^41^. The patients presenting the most reduced ME showed greater ventilatory inefficiency, likely negatively affecting the VE and perfusion in the lungs and muscles, thus decreasing exercise capacity^41^. This was not corroborated in the healthy women at an intensity of the VT_1_.

Rinaldo et al. did not find significant differences between patients with preserved and those with decreased exercise capacity in ventilatory efficiency^35^. The authors concluded that COVID-19 survivors showed a mild reduction in their exercise capacity without relevant functional sequelae in the ventilatory and gas exchange response to exercise after 3 months. The reduction in exercise capacity was probably caused by muscle deconditioning. Unlike our study, these authors did not analyze ME. We likely found that patients with decreased ME showed lower ventilatory efficiency due to the timing of the tests (6–8 weeks after the patients recovered from COVID-19). Three months could be enough time to recover ventilatory efficiency in COVID-19 survivors with decreased exercise capacity^35^ or impaired ME. Maybe, female patients with COVID-19 could recover pulmonary function before muscle function in the medium-long term. More studies are needed to confirm these hypotheses.

Although ventilatory efficiency was lower in patients recovered from COVID-19 compared to the group of healthy women, the values of the VE∙VCO_2_^–1^ slope could not be considered pathological in patients recovered from COVID-19^35^. VE∙VCO_2_^–1^ slope values greater than 34 are used as a reference to determine important deterioration in several diseases^13^.

It was found that the ME and ventilatory efficiency during exercise declined in patients recovered from COVID-19, subsequently producing a hypothetical greater oxygen cost in the respiratory process probably directed, at least in part, by an increase in the VO_2SC_^32^. While it appears that adult females recovered from COVID-19 have increased VO_2SC_ and impaired ME and ventilatory efficiency, further studies are needed to establish a possible physiological causal relationship between the kinetics of VO_2_, ME and ventilatory efficiency in female patients recovered from SARS-CoV-2.

Finally, FVC (%) and FEV (%) were significantly lower in female patients recovered from COVID-19 than in healthy female group. Although spirometry values of FVC (%) and FEV (%) were not considered pathological in female patients recovered from COVID-19, they could be related to complications of COVID-19 pneumonia. In addition, BMI in the COVID-19 group (overweight) was also significantly higher than in the control group (normal weight). This could also condition the results during CPX in relation to ME, ventilatory efficiency and the VO_2sc_.

Several limitations should be considered. The sample size was small. The group of women recovered from COVID-19 was a heterogeneous group consisting of patients who were hospitalized, patients admitted to the intensive care unit, and patients who were not hospitalized. The degree of severity of the SARS-CoV-2 disease (hospitalized vs. non-hospitalized) could have influenced the interpretation of the results. More studies analyzing the impact of hospitalization, intensive care unit and non-hospitalization on the VO_2sc_, ME and ventilatory efficiency in COVID-19 survivors will be needed to substantiate such claims.

## Conclusions

Female patients recovered from SARS-CoV-2 had significant pulmonary and muscle dysfunction compared with healthy women of similar characteristics. These findings were corroborated by detecting an increase in the VO_2sc_ and a deteriorated ME and ventilatory efficiency during the constant-load test.

Strength and endurance rehabilitation programs should be considered during the early phase of recovery in patients recovered from SARS-CoV-2.

## Data Availability

All data of this study are available from the corresponding author on reasonable request.
